# Acute modulation of common synaptic inputs and motor unit discharge rates following neuromuscular electrical stimulation superimposed onto voluntary contractions

**DOI:** 10.3389/fphys.2025.1590949

**Published:** 2025-07-18

**Authors:** R. Borzuola, S. Nuccio, A. Del Vecchio, I. Bazzucchi, F. Felici, G. De Vito, A. Macaluso

**Affiliations:** ^1^Department of Movement, Human, and Health Sciences, University of Rome “Foro Italico”, Rome, Italy; ^2^Department Artificial Intelligence in Biomedical Engineering, Faculty of Engineering, Zentralinstitut für Medizintechnik (ZIMT), Friedrich-Alexander University Erlangen-Nürnberg, Erlangen, Germany; ^3^Department of Biomedical Sciences, University of Padova, Padova, Italy

**Keywords:** electrical stimulation, motor unit, common synaptic input, force steadiness, neural drive, coherence analysis, HDsEMG

## Abstract

**Introduction:**

Superimposing neuromuscular electrical stimulation (NMES) onto voluntary contractions induces specific neuro-physiological adaptations that may have a direct effect on force related outcomes. This study investigated motor unit discharge characteristics and force steadiness following three acute experimental conditions: NMES superimposed onto isometric contractions (NMES + ISO), passive NMES, and isometric contractions only (ISO).

**Methods:**

Seventeen healthy volunteers participated in the study. Each condition involved 20 intermittent (6s contraction/6s rest) isometric ankle dorsi flexions at 20% of their maximum voluntary contraction (MVIC). NMES was delivered to the tibialis anterior (TA) during NMES and NMES + ISO. High-density surface electromyography (HDsEMG) was used to record myoelectric activity in the TA during steady force-matching contractions, at 10% MVIC, performed immediately after each experimental condition. Motor unit discharge rate (DR) and inter-spike variability (ISIvar) were analyzed from decomposed HDsEMG signals. Coherence analysis was performed to evaluate the strength of common synaptic input across different frequency bands and the proportion of common synaptic input (pCSI) received by spinal motoneurons. Force steadiness was evaluated using the coefficient of variation of force (Force_CoV_).

**Results:**

NMES + ISO significantly increased motor unit DR compared to baseline and post-intervention NMES. NMES + ISO also induced an increase in pCSI compared to baseline, ISO and NMES. Force_CoV_ was reduced after NMES + ISO compared to all experimental conditions, indicating improved force steadiness.

**Discussion:**

These results suggest that superimposing NMES onto voluntary contractions can enhance motor unit firing rate and pCSI at low force levels. These adaptations seem to positively contribute to force steadiness, likely by engaging filtering mechanisms which minimize the independent synaptic noise affecting motor control. These findings provide new perspectives on the adaptations induced by NMES exercise, highlighting some of the neuro-physiological mechanisms involved and enriching our knowledge of how the neuromuscular system responds and adapts to NMES-based interventions.

## Introduction

Neuromuscular electrical stimulation (NMES) has proven to be highly effective in maintaining, restoring, and improving muscle mass and function in both healthy individuals and those with injuries (Vanderthommen and Duchateau, 2007; Maffiuletti, 2010). In rehabilitation settings, NMES can assist in recovering muscle function and motor control following injury or surgery, facilitating the relearning of effective muscle force generation and modulation capabilities (Herzig et al., 2015; Labanca et al., 2018; 2022). In sports training, NMES can complement conventional strength training methods, offering a targeted approach to enhance force control in specific muscle groups (Filipovic et al., 2011). Notably, superimposing NMES onto voluntary muscle contractions (NMES+) has yielded even greater improvements in muscle function and performance than NMES alone, due to specific neurophysiological adaptations (Paillard, 2018; Borzuola et al., 2023a). Recent studies exhibited that NMES+ has a facilitatory effect on both spinal and cortical responses (Lagerquist et al., 2012; Borzuola et al., 2020; 2023c; 2024; Scalia et al., 2023) compared to passive NMES and voluntary contractions alone, indicating significant modulation of these pathways through both peripheral and central mechanisms. However, these studies involved the assessment of evoked responses, which do not reflect changes in volitional neural activity.

Volitional neural activity could be achieved non-invasively by evaluating the behavior of large populations of motor units (MUs) through decomposition of high-density surface electromyography (HDsEMG) (Holobar and Zazula, 2007; Merletti et al., 2008). Several studies indicated that this technique allows for a reliable measurement of MUs discharge characteristics and estimation of the synaptic input received by spinal motor neurons (Del Vecchio et al., 2019b). Interestingly, a recent study carried out by our research group exhibited that short bouts of NMES+ can acutely increase MU firing rates at high force levels, suggesting an enhanced neural drive which could positively impact force generating capacities and control (Borzuola et al., 2023b). However, the impact of NMES+ on MU behavior as well as on the shared inputs received by motor neurons during low-force contractions, where coordinated force production is required, remains unclear.

The generation and control of muscle force rely on the activation of spinal motor neurons, which integrate a mix of excitatory and inhibitory signals originating from spinal, supraspinal, and sensory pathways. These signals include both independent and common synaptic inputs to motor neurons, although, notably, during tasks requiring force control (i.e., force-matching tasks), it is the common component within the low-frequency band (<5 Hz) that predominantly controls force generation (Farina and Negro, 2015; Castronovo et al., 2018). Coherence analysis allows to evaluate the correlations in MU spike trains within (intramuscular coherence - IMC) and between (intermuscular coherence) muscles in the frequency domain and can be used to estimate the strength of the common synaptic input (CSI) in specific frequency bandwidths such as delta (0–5 Hz), alpha (6–12 Hz) and beta (15–35 Hz) (Castronovo et al., 2018). Peak coherence values within these bands are typically analyzed to infer the level of shared inputs that modulate various aspects of neuromuscular function such as force control (delta), afferent and spinal circuits (alpha) and corticospinal pathways (beta) (Castronovo et al., 2015). Force control is inherently linked to the proportion of common synaptic input (pCSI) received by spinal motoneurons (i.e., proportion between the common inputs to the motor neurons with respect to the independent synaptic inputs at low frequencies) as it influences their collective behavior, contributing to coordinated muscle contractions and force steadiness (Negro et al., 2009; Hug et al., 2023). Thus, an increase in pCSI suggests a higher level of synchronization among motor units, which can reduce synaptic independent noise likely improving the precision of force control (Farina and Negro, 2015).

The effect of NMES on force control is multifaceted and depends on various factors, including stimulation parameters, muscle characteristics, and individual responsiveness. Research indicates that NMES can lead to improvements in force production, endurance, and coordination, thereby enhancing force control capacities (Bezerra et al., 2011). These enhancements are attributed to several underlying mechanisms, including neural adaptations, muscle hypertrophy, and changes in motor unit recruitment patterns (Maffiuletti et al., 2006). Neural adaptations induced by NMES+ involve modifications in motor neuron excitability, synaptic efficacy, and intermuscular coordination (Borzuola et al., 2023a). A recent study which investigated the effect a combination of electrical stimulation and vibration (noise stimulation) on neural plasticity, reported an improvement in motor control strategies and as well as in the ability to regulate force output accurately (Chou et al., 2023). Nevertheless, while previous work has examined MU discharge characteristics following NMES at moderate to high contraction levels, no study has yet evaluated the effects of NMES+ on common synaptic input and force steadiness at low contraction levels, where shared synaptic inputs are particularly relevant.

Therefore, the present study aims to investigate the acute effects of NMES+ on motor unit firing patterns, the proportion and strength of common synaptic inputs to spinal motoneurons, and force steadiness, in comparison to passive NMES and voluntary isometric contractions alone (ISO). Based on the findings of our previous study we hypothesized that NMES + ISO would result in an enhancement of MU discharge rate (DR), pCSI and IMC in the delta band greater than in the other two conditions with a concomitant improvement in force steadiness. By elucidating the relationship between force control and pCSI, this study aims to provide critical insights into the acute neuromuscular responses that are induced by NMES+.

## Methods

### Participants

Seventeen young healthy volunteers (12 males and 5 females, mean ± SD age: 28 ± 5 years, mass: 71 ± 10 kg, height: 1.75 ± 0.7 m), with no history of neurological or orthopedic disorders, participated in the study. A statistical power analysis was performed *a priori*, using a repeated measures approach (ANOVA: Repeated measures, within factors), to determine the sample size (G*Power software version 3.1.9.4; α = 0.05, power = 0.85, effect size = 0.35, sample size = 14) as indicated by Cohen (1992) based on previous works investigating motor unit discharge properties following exercise (Semmler et al., 2004; Borzuola et al., 2023b; Lecce et al., 2023; Nuccio et al., 2024). Specifically, mean motor unit discharge served as primary outcome measure for the power analysis, with pooled effect size estimated from these studies. The participants did not have any experience with NMES exercise before performing the experimental session and were asked not to engage in any strenuous activity 24 h before the experimental session. All procedures were approved by the Internal Review Board of the University of Rome “Foro Italico” (CAR 86/2021) and adhered to the standards defined by the *Declaration of Helsinki*. Written informed consent was obtained from all participants before their first experimental session.

### Experimental procedures

**FIGURE 1:**
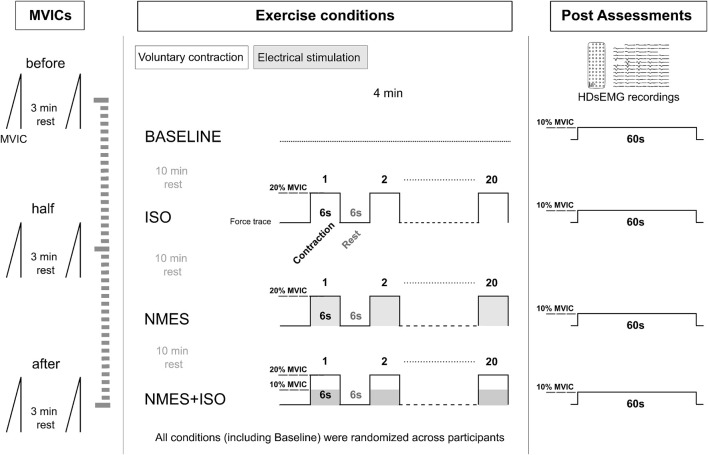
Experimental protocol

The experimental session (Figure 1) lasted about 2 h and consisted of three experimental conditions: (a) NMES applied on the tibialis anterior muscle (NMES) (b) NMES superimposed onto voluntary isometric contraction of the ankle dorsi flexor muscles (NMES + ISO) and (c) voluntary isometric contraction of the ankle dorsi flexor muscles only (ISO). In addition, participants underwent a baseline resting condition, during which they did not perform any exercise. Experimental conditions, including baseline were administered in random order. During each exercise condition participants performed 20 intermittent contractions at 20% of maximal voluntary isometric contraction (MVIC) (6 s contraction/6 s rest), for a total duration of 4 min. The number and duration of these contractions was chosen to prevent the onset of muscle fatigue (Neyroud et al., 2014; Grosprêtre et al., 2018), while at the same time promoting neural adjustments as indicated in previous research (Lagerquist et al., 2012; Borzuola et al., 2020; Borzuola et al., 2023c). During baseline, participants remained seated at rest for 4 min. A recovery interval of 10 min was provided between each experimental condition to minimize carryover effects. All the procedures were conducted on participants’ dominant limb which was determined as the limb preferred for hopping or kicking a ball (Holmbäck et al., 2003). Following each condition, participants performed steady force-matching contractions at 10% MVIC while we assessed tibialis anterior muscle myoelectrical activity with high-density surface EMG (HDsEMG). Real-time visual feedback of both the force output and the expected trajectory were provided to participants at a constant visual gain. MVIC assessments were repeated at three separate times (before, at half and at the end of the experimental conditions) to monitor if any fatigue had arisen throughout the protocol.

### Force recordings and analysis

During the experimental procedures, participants sat comfortably in a chair that was firmly fixed to an adjustable ankle dynamometer (OT Bioelettronica, Turin, Italy). The setup ensured that knee and hip joints were maintained at 90° flexion (with 0° representing full extension and a neutral position, respectively), and the ankle was positioned at 0° of ankle plantar-dorsi flexion (0° = foot orthogonal to the shank axis). The ankle and the foot were firmly strapped to the footplate of the dynamometer using padded Velcro straps to minimize movement, while the non-dominant leg rested on a footrest. The foot plate was connected to a calibrated force transducer (CCT Transducer, Turin, Italy), which was mounted perpendicularly beneath the footplate. The analogue signal from the transducer was amplified with a 500 gain, sampled at 2,000 Hz, and converted to digital data by a 16-bit external analogue-to-digital converter (Sessantaquattro, OT Bioelettronica, Turin, Italy). Force and HDsEMG signals were acquired with the software OTbiolab (OT Bioelettronica, Turin, Italy). Prior to maximal testing, participants completed a warm-up and familiarization protocol involving 20 submaximal isometric dorsiflexions (about 50% of perceived maximal contraction). The MVIC assessment consisted of progressively increasing the force of the dorsi flexor muscles from zero to a maximum over 3 s and maintaining the maximal value for ∼3 s before relaxing. Participants were given verbal encouragement to promote their maximal effort. Two MVIC attempts were performed, each spaced 3 min apart to minimize the effect of fatigue. MVIC was chosen as the largest 500 ms average achieved within a force recording. Assessment of MVIC was then used to define a target isometric dorsi flexion force as 20% MVIC, which was used for the exercise conditions, and the steady force level at 10% MVIC. The steady force-matching contractions at 10% MVIC had a duration of 60 s. The magnitude of the force fluctuations around the target force was quantified by computing the coefficient of variation for force (Force_CoV_: ratio of standard deviation relative to the mean). A moving 20 s window (100 ms increments) was used to identify the steadiest 20 s (lowest coefficient Force_CoV_) of each 60 s trial (Tvrdy et al., 2025).

### NMES

**FIGURE 2:**
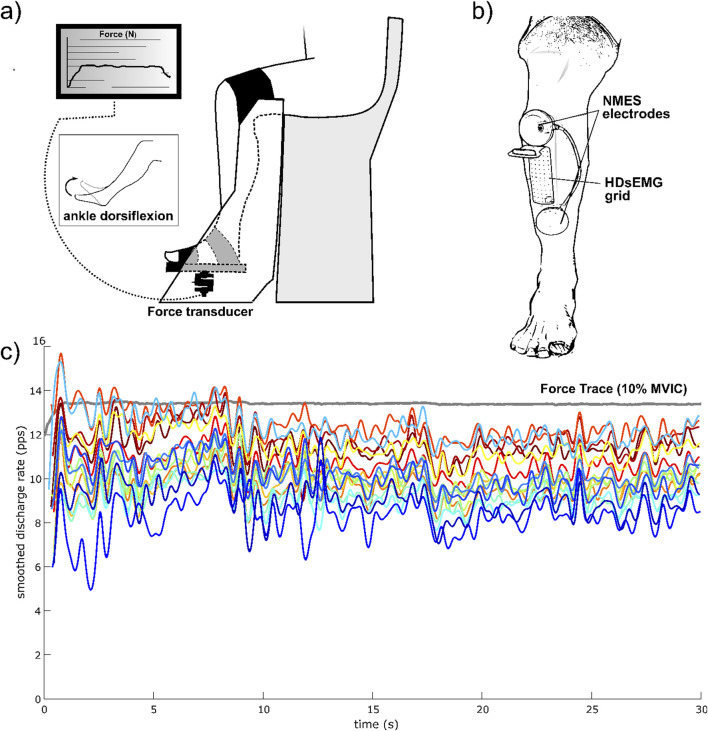
Experimental setup and HDsEMG recording. **(a)** Participants’ position on the dynamometer; **(b)** NMES electrodes and HDsEMG positioning on the TA muscle; **(c)** Example of smoothed discharge rate of all identified MUs of one trial, from one participant, during the steady force-matching isometric contraction at 10% MVIC.

NMES was delivered over the TA muscle using a portable muscle electrical stimulator (Chattanooga Wireless Professional, DJO Global, Vista, CA, United States). NMES was applied either passively or superimposed onto voluntary contraction of the dorsi flexor muscle. The stimulator produced a rectangular, balanced biphasic pulse and was constantly handled and controlled by the operator. Two self-adhesive electrodes (Compex Dura-Stick plus, 50 × 50 mm, DJO Global, Vista, CA, United States) were used to deliver the stimulation. The anode was placed over the motor point of the tibialis muscle, while the cathode was placed distally on the same muscle, about 6 cm from the anode, as illustrated in Figure 2. The motor point of the tibialis anterior muscle was identified at the beginning of the experimental session with a hand-held anode ball electrode in accordance with the electrical stimulator user’s guide. NMES was administered with a pulse frequency of 50 Hz and a pulse width of 400 μs per phase to generate higher forces while promoting the highest comfort during electrical stimulation, as reported in previous investigations (Baldwin et al., 2006; Wiest et al., 2017). The current pulse intensity of the stimulation was manually adjusted in accordance with each participant’s tolerance. Before the beginning of the experimental conditions, participants familiarized with the electrical stimuli for about 10 min at low intensity. In the NMES condition, current pulse intensity was increased (average NMES intensity: 20.4 mA; range 10.5–32.2 mA) until the passively stimulated ankle dorsi flexion reached the target force at 20% MVIC. During ISO, participants were required to match the target force of 20% MVIC by voluntarily contracting their dorsi flexor muscles. During NMES + ISO, current pulse intensity was set to produce half of the target force (10% MVIC; average NMES intensity: 15.1 mA; range 8.6–26.9 mA) and participants were asked to voluntarily contract their dorsi flexor muscles to achieve the full target of 20% MVIC. In this condition, participants were asked to relax their tibialis anterior muscle before the first and after the 10th contraction while the investigator adjusted the current intensity to make sure that the force produced by the stimulator alone corresponded to half of the target force throughout the entire NMES + ISO condition.

### HDsEMG recordings

HDsEMG signals were acquired in monopolar derivation from the TA muscle using a high-density adhesive grid of 13 × 5 equally spaced electrodes (gold-coated, 1 mm diameter, 8 mm inter-electrode distance (IED); GR08MM1305, OT Bioelettronica, Turin, Italy). A trained operator first located the perimeter of the muscle by manual palpation and, then, positioned the grid over the most innervated areas of the distal area of the TA muscle (Figure 2), as indicated in previous works (Del Vecchio et al., 2019a; Casolo et al., 2020; Borzuola et al., 2023b). The area of the skin where the grid was applied was shaved, lightly abraded, and cleansed with 65% ethanol to optimize electrode-skin conductivity. The grid was applied over the skin of tibialis anterior muscle parallel to the lateral margin of the tibia with a bi-adhesive foam layer designed to match the HDsEMG grid (SpesMedica, Battipaglia, Italy). The cavities of the foam layer were filled with conductive paste (SpesMedica) to obtain skin-electrode contact. The reference electrode of the grid was positioned on the medial malleolus of the tested leg with a moistened ankle strap while the electrode grid was connected to a multichannel amplifier (Sessantaquattro, OT Bioelettronica, Turin, Italy).

The HDsEMG signals were sampled at 2,000 Hz, band-pass filtered (3 dB bandwidth, 10–500 Hz), converted to digital data by the multichannel amplifier, and visually inspected to detect and exclude channels with noise. On average, 2.6 ± 1.1 channels per grid were excluded before decomposition.

### Motor unit decomposition and analysis

Then, HDsEMG signals were decomposed into individual MU discharge times using a validated convolutive blind source separation method (Holobar and Zazula, 2007), which was implemented in a MATLAB (R2022a; The MathWorks, Natick, MA) tool (v. 4.9; DEMUSE; The University of Maribor, Slovenia). The discharge times of individual MUs were then converted into binary spike-trains, which were then visually inspected by the investigator to remove MUs showing a pulse-to-noise ratio (PNR) ≤30 dB or an inter-spike time interval higher than 2 s (Holobar and Farina, 2014). MUs were tracked across conditions using a widely validated procedure (Martinez-Valdes et al., 2017). To examine the discharge characteristics of the identified MUs the average MU discharge rate (DR) and the variability for the Inter-spike Interval (ISI_var_) were estimated during the 20 s time window of the steady force-matching contraction that was selected for computing the Force_CoV_.

#### Intramuscular coherence (IMC)

A coherence analysis was performed on cumulative spike trains (i.e., index of the neural drive to the muscle; CSTs). Specifically, intramuscular coherence (IMC) was estimated by cross-correlating CSTs from increasing groups of MUs (e.g., eight identified MUs were pooled within two groups including up to four MUs each) using a Welsh periodogram with non-overlapping 1 s Hanning windows. The number of MUs in each of the two groups varied from 1 to the maximum number (half of the total number of identified MUs). One hundred random permutations of the identified MUs were performed for each iteration, and the average of all permutations was extracted and used for further analysis. The coherence profile was estimated within the full-frequency bandwidth (Farina et al., 2017). The coherence in each frequency band was estimated as the integral of the coherence estimates within delta (1–5 Hz), alpha (6–12 Hz), and beta (15–30 Hz) bands. The average coherence in the frequency range 100–250 Hz was set as the *bias* level and therefore subtracted from the analyses of coherence profiles (Del Vecchio et al., 2019b). To compare IMC across limbs and groups, we considered a minimum of six MUs for each submaximal isometric task. All seventeen subjects had at least six matched Mus for each isometric task. The Fisher “z-transform” was applied to the coherence estimates C(f) to obtain a normally distributed variable Z(f), as illustrated in Equation 1 (Halliday and Rosenberg, 2000).

$$Z\left(f\right)=\sqrt{2n}\;\tanh^{-1}\sqrt{C\left(f\right)}$$ (1)

Where *n* represents the number of time segments that were used for the analysis.

#### pCSI

The proportion of common synaptic input (pCSI) received by spinal motoneurons was estimated by computing the slope (rate of change) of the relation between average coherence values in the delta band and the number of identified MUs (Farina et al., 2017). This analysis was performed on the low-frequency delta bandwidth (1–5 Hz), as it has been shown to be the main determinant of the force output (Farina et al., 2017). The pCSI reflects the fraction of the total input that is shared between motoneurons and that is unrelated with the independent components of the synaptic input.

### Statistical analysis

Statistical analysis was performed using IBM SPSS 24.0 (IBM Corp., Armonk, NY, United States) and Jamovi 2.2.5 (The Jamovi project, Sydney, Australia). The Shapiro-Wilk test was used to assess the normality of distribution of the reported variables. When variables did not show a normal distribution, these were log-transformed to meet the assumption of normality before applying the statistical test. To analyze differences in DR and ISIvar of MUs, separate linear mixed-effects models (GAMLj pack: General Analyses for the Linear Model in Jamovi) were used to account for the hierarchical structure of the data (Tenan et al., 2014; Héroux, 2021). Condition (Baseline, ISO, NMES, NMES + ISO) was considered as a fixed effect while participant-specific variability was modeled with random intercepts, and restricted maximum likelihood estimation (REML) was applied to fit the models. The analysis was performed using only tracked MUs which were identified in all four conditions. Significance of the fixed effect was assessed by an F-test using Satterthwhaite’s approximation for the degrees of freedom. A Bonferroni-Holm correction was applied to account for multiple comparisons. A one-way repeated measurement ANOVA was used to detect differences in Force_CoV_, pCSI, and IMC within the delta, alpha, and beta bands between conditions (Baseline, ISO, NMES, NMES + ISO). The same analysis was performed to evaluate differences between the three MVIC assessments (before, half and end). The Mauchly’s test was used to assess sphericity of the analyzed variables, and the Greenhouse-Geisser correction was applied if sphericity was violated. A Bonferroni-Holm correction was applied when needed to account for multiple comparisons. In addition, to investigate the relationship between Force CoV (dependent variable) and pCSI, DR, ISIvar, and IMC in the different frequency bands, linear mixed-effects models were used. These models included subject as a random effect (intercept) to account for repeated measures design. Model fit and power were evaluated using marginal *R* ^2^ (variance explained by fixed effects) and conditional *R* ^2^ (variance explained by both fixed and random effects). Residual plots were inspected to assess assumptions of linearity and homoscedasticity. For all statistical tests, the significance level was set to 0.05. Data are reported as means ± SD unless stated elsewhere.

## Results

### MVIC and force steadiness

The repeated measures ANOVA revealed no significant differences between the MVIC assessments (before: 222 ± 81 N; half: 219 ± 78 N; end: 220 ± 82 N) suggesting that no fatigue had arisen throughout the experimental protocol.

**FIGURE 3:**
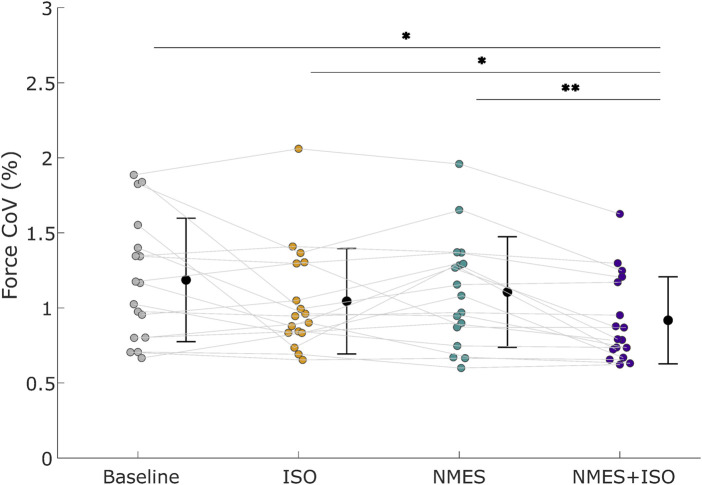
Force steadiness. Force coefficient of variation (Force_CoV_) across different conditions. Black circles and error bars indicate means ± SD. Individual participant data (*n* = 17 participants) are presented as different color dots for each experimental condition. Mean values are represented by a black dot with SD black bars. *P < 0.05, **P < 0.01.

The repeated measures ANOVA indicated a significant effect of Condition (F = 5.34; η_p_^2^ = 0.25; P = 0.003). Post-hoc tests showed a decrease in Force_CoV_ following NMES + ISO (0.93% ± 0.30%) compared to baseline (1.19% ± 0.41%; −21.8%; *d* = 0.66; P = 0.013; Confidence Interval (CI) [−0.393, −0.109]), ISO (1.04% ± 0.35%; - 10.6%; *d* = 0.35; P = 0.02; CI [−0.199, −0.019]) and NMES (1.12% ± 0.46%; −16.9%; d ≈ 0.55; P = 0.001; CI [−0.333, −0.045]). Data are illustrated in Figure 3 and reported in Table 1.

**TABLE 1:** Data and statistical results from force and electromyographic recordings.

	Force_COV (%)_	DR (pps)	ISIvar (%)	IMC (z-score)			pCSI
				Delta	Alpha	Beta	
Baseline	1.19 ± 0.41	10.09 ± 0.98	10.55 ± 2.02	6.05 ± 0.98	2.44 ± 1.29	1.48 ± 0.95	0.44 ± 0.13
ISO	1.04 ± 0.35	10.44 ± 1.10	10.90 ± 1.45	6.37 ± 0.95	2.65 ± 1.08	1.66 ± 0.67	0.46 ± 0.13
NMES	1.12 ± 0.46	10.38 ± 1.24	11.00 ± 2.14	6.07 ± 1.21	2.42 ± 1.18	1.56 ± 0.79	0.44 ± 0.14
NMES + ISO	0.93 ± 0.31	10.51 ± 1.02	11.36 ± 3.11	6.37 ± 1.03	2.42 ± 1.09	1.53 ± 0.56	0.50 ± 0.14
Baseline vs. ISO (P-value)	(0.09)	**(0.002)**	(0.98)	(0.11)	(0.15)	(0.12)	(0.22)
Baseline vs. NMES (P-value)	(0.42)	(0.06)	(0.30)	(0.94)	(0.89)	(0.58)	(0.86)
Baseline vs. NMES + ISO (P-value)	**(0.01)**	**(0.001)**	(0.83)	(0.12)	(0.85)	(0.50)	**(0.001)**
ISO vs. NMES (P-value)	(0.19)	(0.28)	(0.31)	(0.21)	(0.14)	(0.29)	(0.33)
ISO vs. NMES + ISO (P-value)	**(0.02)**	(0.26)	(0.85)	(0.99)	(0.08)	(0.18)	**(0.03)**
NMES vs. NMES + ISO (P-value)	**(0.001)**	**(0.03)**	(0.42)	(0.09)	(0.98)	(0.18)	**(0.02)**

Data are presented as group means ± standard deviation. In bold statistically significant differences between conditions.

### Motor unit properties

#### Number of identified MUs

A total of 1009 MUs were identified in the TA with an average of 59.3 ± 14.8 MUs per participant. Of all MUs, 220 MUs (about 22%) were tracked across all four conditions (Baseline, ISO, NMES, NMES + ISO), with an average number of 12.9 ± 3.3 tracked MUs per participant.

#### DR and ISIvar

**FIGURE 4:**
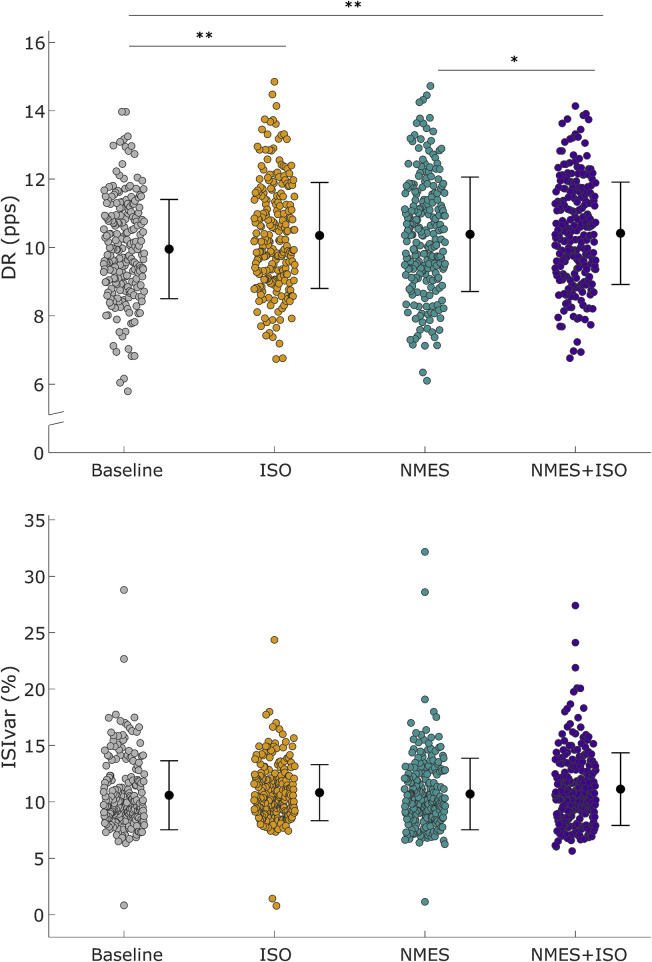
Differences in motor unit discharge rate (DR) and inter-spike variability (ISIvar). Upper panel: Distribution of DR of all MUs that were identified in the TA muscle. DR values are represented by using different color-filled circles for each experimental condition. Lower panel: Distribution of ISIvar of all identified MUs. ISIvar values are represented using different color-filled circles for each experimental condition. Mean values are represented by a black dot with SD black bars. *P < 0.05, **P < 0.01.

The fixed-effects omnibus test indicated a significant effect of Condition (F = 9.52; P < 0.001) on MU DR. Post-hoc analysis showed that MU DR was significantly increased following NMES + ISO (10.51 ± 1.02 pps) compared to both baseline (10.09 ± 0.98 pps, +4.2%, *d* = 0.52, P = 0.001, CI [0.365, 0.810]) and NMES (10.38 ± 1.24 pps, +1.25%, *d* = 0.26, P = 0.038, CI [0.022, 0.562]). Moreover, MU DR was significantly greater after ISO (10.44 ± 1.10 pps) compared to baseline (10.09 ± 0.98 pps, +3.5%, *d* = 0.37, P = 0.002, CI [0.193, 0.637]). There was no significant main effect of Condition (F = 0.47; P = 0.701) on ISIvar. Data are illustrated in Figure 4 and reported in Table 1.

### IMC

**FIGURE 5:**
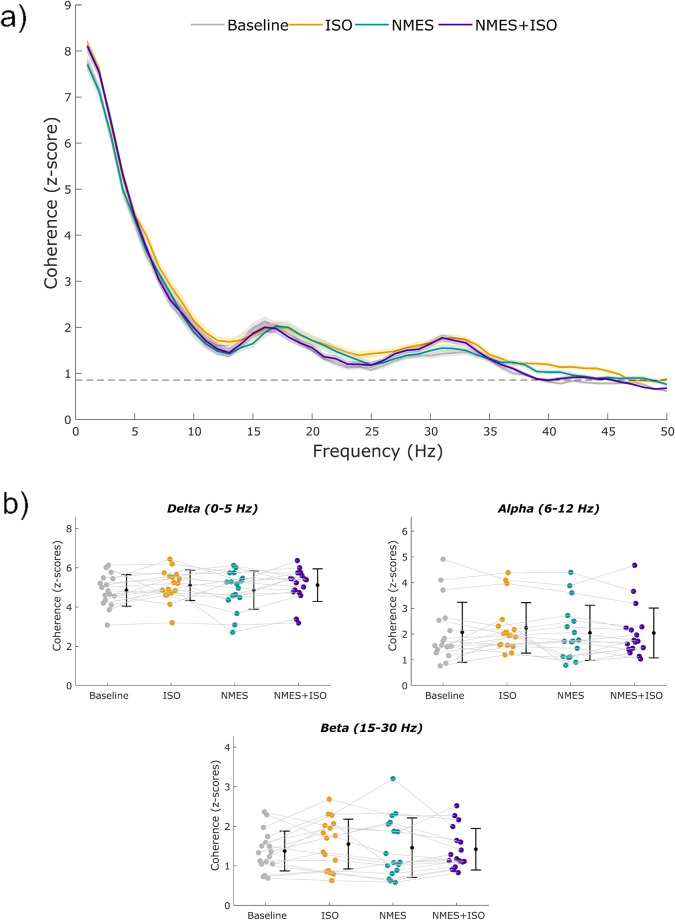
Profiles of intramuscular coherence (IMC) and IMC across frequency bands. **(a)** Mean IMC across MUs of the TA during a sustained contraction at 10% MVIC. The shaded areas represent the standard error of the mean. **(b)** Individual values of intramuscular coherence within the delta (1–5 Hz), alpha (6–12 Hz), and beta (15–30 Hz) bands. Participant-specific values are represented using color-filled circles for each experimental condition. Mean coherence values are represented by a black dot with SD black bars.

Profiles of intramuscular coherence for each experimental condition are illustrated in Figure 5. When analyzing IMC within each frequency band, no significant differences emerged for any frequency band following the experimental conditions. Data are reported in Table 1.

### pCSI

**FIGURE 6:**
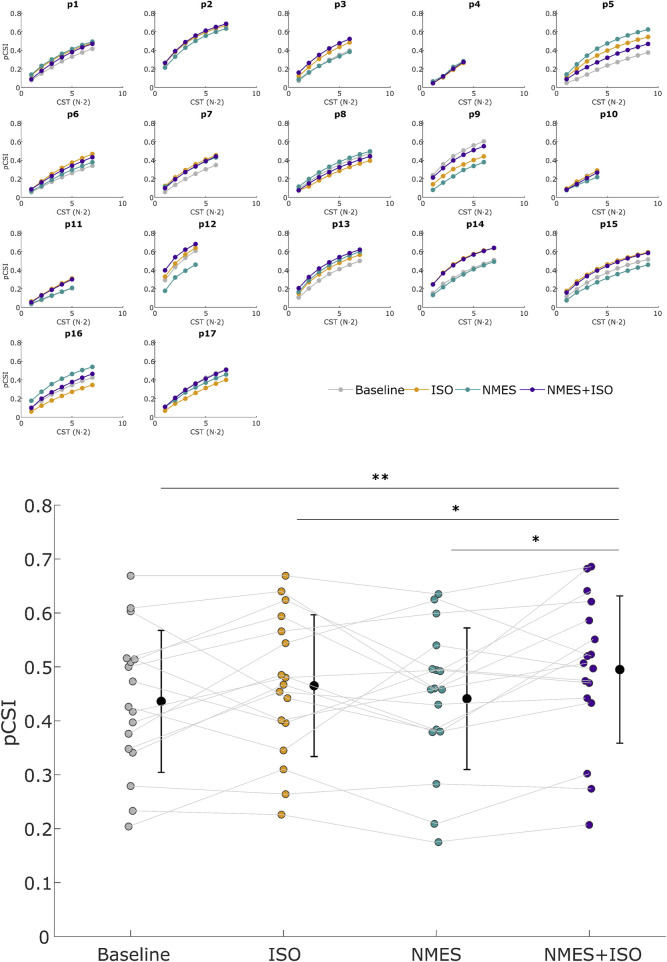
Proportion of common synaptic input (pCSI). Upper panel: pCSI of all subjects across all conditions. pCSI was derived from coherence between Cumulative Spike Trains (CST), with CST (N·2) referring to the number of pairwise combinations of MUs spike trains used for the calculation (N = number of MUs). Lower panel: Mean pCSI of each experimental condition. Participant-specific values are represented using color-filled circles for each experimental condition. Mean pCSI values are represented by a black dot with SD black bars. *P < 0.05, **P < 0.01.

There was a significant effect of Condition (F = 3.39; η_p_^2^ = 0.18; P = 0.025) for pCSI. Post-hoc analyses showed a significantly higher pCSI of the TA muscle after the NMES + ISO condition (0.50 ± 0.14) compared to baseline (0.44 ± 0.13; *d* = 0.45, P = 0.001, CI [0.028, 0.090]), ISO (0.46 ± 0.13; *d* = 0.22, P = 0.037, CI [0.002, 0.058]) and NMES (0.44 ± 0.13; *d* = 0.40, P = 0.020, CI [0.010, 0.098) conditions (Figure 6).

### Linear regression analysis

When evaluating the relationship between pCSI and Force_CoV_, the linear mixed-effects model revealed a statistically significant model (F(1,42.9) = 16.40, P < 0.001). The marginal *R* ^2^ was 0.26 suggesting that pCSI accounts for approximately 26% of the variance in Force_CoV_, while the while the conditional *R* ^2^ was 0.61, reflecting additional variance explained by random effects. The regression equation was Y = −1.31*X + 1.07 + ε. The 95% CI for the slope ranged from −2.07 to −0.72, suggesting that for each unit of increase in pCSI, Force_CoV_ decreases by about 0.72–2.07 units.

**FIGURE 7:**
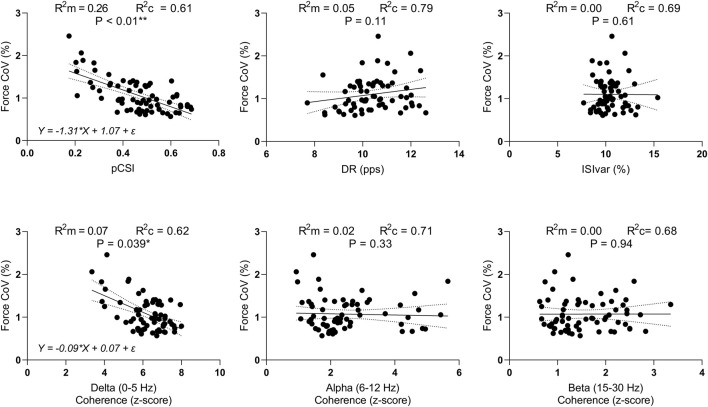
Linear mixed-effects models. Scatterplots of Force_CoV_ in function of pCSI, DR, ISIvar, and IMC in the delta, alpha, and beta bands. R^2^m = marginal R-squared; R^2^c = conditional R-squared *P < 0.05, **P < 0.01.

When considering the relationship between IMC in the delta band and Force_CoV_, the analysis revealed a statistically significant model (F(1,66.6) = 4.44, P = 0.039). The marginal *R* ^2^ was 0.07 and the conditional *R* ^2^ was 0.62. The regression equation was Y = −0.09*X + 0.07 + ε, while the 95% CI for the slope ranged from −0.18 to −0.01 suggesting that, for each unit of increase in IMC in the delta band, Force_CoV_ decreases by about 0.01–0.18 units. None of the other independent variables that were evaluated exhibited a statistically significant linear mixed-effects model. Data are presented in Figure 7.

## Discussion

The main finding of this study was that NMES + ISO significantly increased motor unit discharge rate (DR) in the TA muscle during low-intensity ankle dorsi flexions, compared to baseline levels and passive NMES. Furthermore, pCSI was greater following NMES + ISO compared to baseline, NMES and voluntary isometric contractions alone. Finally, NMES+ led to a reduction in the Force_CoV_ compared to all the experimental conditions, indicating improved force steadiness. No differences between conditions were found in the IMC in different frequency bands. These results partially confirm our hypothesis and highlight the potential of NMES+ to enhance motor unit firing rates and optimize force control, particularly through the modulation of common synaptic inputs.

The significant increase in DR following NMES+ aligns with previous research indicating that NMES can enhance motor unit recruitment and firing rates (Borzuola et al., 2023b), although the present study revealed this change also at lower force levels (i.e. 10% MVIC). This likely results from the combined effects of voluntary effort and superimposed NMES, facilitating greater motor unit activation and leading to a more substantial neural input to the muscles compared to NMES and voluntary contraction alone. The enhanced DR might ensure that MUs fire at an optimal rate to maintain force output, possibly contributing to improved muscle force production and steadiness.

The significant increase in pCSI that was observed in this study reflects the degree of synchronized input received by motor neurons, which is crucial for coordinated muscle contractions and force control (Farina and Negro, 2015). An increased pCSI indicates enhanced synchronization of motor unit discharge, improving the precision and stability of force production. This enhancement might be attributed to the integrated effect of voluntary contraction and electrical stimuli, which is likely to promote the recruitment of additional motor units, thereby increasing overall synaptic input to the motor neuron pool and leading to greater synchronization of motor unit activity. Moreover, some researchers suggested that increased pCSI acts as a filter by attenuating independent synaptic noise (Farina et al., 2014). This mechanism ensures that motor neurons receive consistent and coordinated signals, thus reducing force variability and improving steadiness (Negro et al., 2009). This synchronization is particularly important during low-force tasks, in which small variations in neural input can lead to noticeable fluctuations in force production (Enoka and Farina, 2021).

The reduction in Force_CoV_ following NMES+ indicates a relevant improvement in force steadiness, which is critical for performing precise motor tasks (Enoka and Duchateau, 2017). The improved force steadiness can be explained by an enhanced synchronization of motor unit activity, whereas increased pCSI leads to coordinated firing of motor units, reducing variability in force production (Farina and Negro, 2015). This association was confirmed by the linear mixed-effects model analysis that was conducted in the present study, which revealed a moderate relationship between Force_CoV_ and pCSI. As previously discussed, the recruitment of additional motor units during NMES+ may further contribute to force steadiness by distributing the workload across a larger pool of motor units, reducing the relative load on individual motor units and enhancing overall muscle performance (Gregory and Bickel, 2005).

The IMC within the delta, alpha and beta bands were similar following the four experimental conditions. The absence of differences in the delta band does not agree with our hypothesis as an increase in the strength of the common synaptic input in the low frequency band was expected, with a similar trend as pCSI. However, while both pCSI and IMC in the delta bands both measure aspects of motor unit synchronization, each of these variables focuses on different mechanisms of neural control. pCSI represents the proportion of input that motor neurons receive from common sources, reflecting the degree of shared synaptic input among motor neuron, whereas IMC in the delta band highlights low-frequency coordination of motor units but may not directly indicate the source or proportion of synaptic input (Del Vecchio et al., 2023; Hug et al., 2023). The observed increase in pCSI with no change in delta band IMC in this study suggests that NMES + ISO enhances force steadiness through increased proportion of common synaptic input with respect to independent synaptic input, rather than altering low-frequency coherence patterns. Nevertheless, we found a weak but significant linear association between Force_CoV_ and IMC in the delta band, reinforcing the established view that increased coherence in low-frequency bandwidths might enhance force steadiness.

Some authors have indicated that neural oscillation in the alpha band are associated with physiological tremor, which can ultimately affect force steadiness and generation capacity (Enoka and Farina, 2021; Lecce et al., 2023; Nuccio et al., 2024). Alteration of afferent inputs and spinal reflex excitability appears to be associated with such involuntary synaptic noise, which characterize alpha oscillation (Nuccio et al., 2024). Nonetheless, despite previous studies which indicated an acute effect of NMES+ on spinal and supraspinal activity (Borzuola et al., 2020; Borzuola et al., 2023c), no acute changes in the IMC in the alpha band emerged after the experimental conditions. This suggests that short bouts of exercise, including NMES+, might not be sufficient to induce the neural plasticity that is required to significantly alter the synchronized neural activity in the alpha band. An alternative explanation might involve the differences in cortico-motoneuronal connectivity between the tibialis anterior (TA) and other muscles (Lauber et al., 2018). For instance, the effects of NMES+ on spinal excitability were commonly evaluated in the soleus muscle (Borzuola et al., 2020; Scalia et al., 2023; Scalia et al., 2024), which primarily relies on spinal mechanisms (Lagerquist et al., 2012). As a result, this muscle might show larger alpha band oscillations compared to the tibialis anterior (TA), which has stronger cortico-motoneuronal connectivity and potentially greater supraspinal control (Lauber et al., 2018).

IMC in the beta bands appears to be linked to corticospinal transmission and motor control (Watanabe and Kohn, 2015). However, some authors indicated that, as muscles act as low-pass filters, beta inputs are thought to have only limited influence on voluntary force production (Farina and Negro, 2015). Specifically, recent evidence revealed that small changes in muscle force can only be determined by bursts of beta activity which, due to its irregular nature, may only have a minimal impact on controlling voluntary force (Zicher et al., 2023). The findings of the present study are in line with these results as no changes in beta IMC was found after the experimental conditions.

The effects of NMES+ on force control and neural drive are mediated by both peripheral and central mechanisms. Peripherally, NMES+ induces muscle contractions through direct stimulation of motor axons (Collins, 2007). This direct activation bypasses the usual voluntary pathways and can lead to greater motor unit recruitment, including the MUs that are not typically recruited during voluntary contractions (Bickel et al., 2011). Centrally, NMES+ can induce plastic changes in the neural pathways involved in motor control (Carson and Buick, 2021). The increased motor neuron excitability and synchronization that were found following NMES+ suggest that this intervention can modulate both spinal and supraspinal pathways. The facilitatory effects on spinal and cortical responses that were observed in previous studies (Borzuola et al., 2020; Borzuola et al., 2023c; Scalia et al., 2023) support this notion. These central adaptations likely contribute to the enhanced neural drive and improved force steadiness as indicated in the present study.

Improvements in force steadiness are crucial in rehabilitation settings for the recovery of fine motor skills, as well as in athletic contexts, where greater force steadiness can translate into improved precision and control during sports activities requiring fine motor coordination. The findings of the present study indicate that NMES+ could significantly enhance neural drive and improve force steadiness making it a valuable tool for rehabilitation and performance enhancement.

While this study provides valuable insights into the neuromuscular adaptations induced by NMES+, further research is needed to elucidate the underlying mechanisms fully. Longitudinal studies investigating the chronic effects of NMES+ on motor unit behavior and muscle performance would provide a more comprehensive understanding of its benefits. Exploring the effects of different NMES parameters, such as frequency, intensity, and duration, could help to optimize NMES protocols for various applications. Investigating the effects of NMES+ in different populations, including older adults and individuals with neuromuscular disorders, would also be valuable, as these populations may significantly benefit from the enhanced neural drive and improved force steadiness associated with NMES+. Moreover, understanding how NMES+ influences muscle function and force control in these groups could inform the development of tailored rehabilitation protocols.

This study has several limitations that should be considered. First, the sample size was relatively small. Although sample size was defined through an appropriate statistical power analysis based on preliminary data and previous study with similar experimental protocols, increasing the number of participants as well as involving different populations (i.e., older adults, individuals with neuromuscular impairments) could support the generalizability of the findings. Second, in this study a relatively low force level was used. The level for force was chosen, as suggested in previous research studies, to improve the accuracy of decomposition (Negro et al., 2009). However, at 10% MVIC it could be argued that only a subset of MUs could be analyzed thus hindering the ability to evaluate coherence and neural drive in a larger cohort of MUs. Finally, the study did not explore the underlying molecular and cellular mechanisms driving the observed neuromuscular adaptations. As suggested by some authors (Al-Majed et al., 2004; Guo et al., 2021), assessing the molecular and cellular changes in muscle and neural tissues in response to NMES could provide deeper insights into the mechanisms driving the observed adaptations, such as how NMES+ influences neuromuscular junction plasticity, axonal integrity, muscle fiber type distribution, and intracellular signaling pathways involved in muscle growth and adaptation. Further investigation into these mechanisms could provide a deeper understanding of how NMES+ influences muscle and neural function at a fundamental level.

In conclusion, this study demonstrates that NMES+ significantly enhances motor unit discharge rate and the proportion of common synaptic input to spinal motoneurons, leading to improved force steadiness. These findings suggest that NMES+ can optimize neural drive and motor unit synchronization, contributing to more efficient and stable force production. The enhanced force control observed following NMES+ has important implications for rehabilitation and performance enhancement, providing a foundation for developing more effective NMES-based interventions. Further research is needed to explore the long-term effects and optimize the application of NMES+ across different populations and settings.

## Data Availability

The raw data supporting the conclusions of this article will be made available by the authors, without undue reservation.
